# Conformational Analysis of Isolated Domains of *Helicobacter pylori* CagA

**DOI:** 10.1371/journal.pone.0079367

**Published:** 2013-11-01

**Authors:** Amanda P. Woon, Abolghasem Tohidpour, Hernan Alonso, Yumiko Saijo-Hamano, Terry Kwok, Anna Roujeinikova

**Affiliations:** 1 Department of Biochemistry and Molecular Biology, Monash University, Clayton, Victoria, Australia; 2 Department of Microbiology, Monash University, Clayton, Victoria, Australia; 3 Graduate School of Frontier Biosciences, Osaka University, Suita, Osaka, Japan; Veterans Affairs Medical Center (111D), United States of America

## Abstract

The CagA protein of *Helicobacter pylori* is associated with increased virulence and gastric cancer risk. CagA is translocated into the host cell by a *H. pylori* type IV secretion system *via* mechanisms that are poorly understood. Translocated CagA interacts with numerous host factors, altering a variety of host signalling pathways. The recently determined crystal structure of C-terminally-truncated CagA indicated the presence of two domains: the smaller, flexible N-terminal domain and the larger, middle domain. In this study, we have investigated the conformation, oligomeric state and stability of the N-terminal, middle and glutamate-proline-isoleucine-tyrosine-alanine (EPIYA)-repeats domains. All three domains are monomeric, suggesting that the multimerisation of CagA observed in infected cells is likely to be mediated not by CagA itself but by its interacting partners. The middle and the C-terminal domains, but not the N-terminal domain, are capable of refolding spontaneously upon heat denaturation, lending support to the hypothesis that unfolded CagA is threaded C-terminus first through the type IV secretion channel with its N-terminal domain, which likely requires interactions with other domains to refold, being threaded last. Our findings also revealed that the C-terminal EPIYA-repeats domain of CagA exists in an intrinsically disordered premolten globule state with regions in PPII conformation - a feature that is shared by many scaffold proteins that bind multiple protein components of signalling pathways. Taken together, these results provide a deeper understanding of the physicochemical properties of CagA that underpin its complex cellular and oncogenic functions.

## Introduction

*Helicobacter pylori* is a Gram negative pathogenic bacterium that infects the stomach tissue of approximately half the world’s population [1] and is associated with different gastric diseases ranging from gastritis and peptic ulcers to adenocarcinoma and mucosa-associated lymphoid tissue (MALT) lymphoma in humans [2-4]. Cytotoxin-associated gene product A (CagA) is a major virulence factor of *H. pylori*. The *cagA* gene belongs to a 40 kb genetic locus called the cytotoxin-associated gene pathogenicity island (*cag*-PAI). Patients infected with *cag*-PAI-positive strains of *H. pylori* have shown a higher prevalence of gastric-related diseases than those infected with *cag*-PAI-negative strains, indicating that *cag*-PAI plays an important role in *H. pylori* pathogenesis [5]. In addition to the *cagA* gene, *cag*-PAI contains 28-30 genes that encode for the components of a type IV secretion system (T4SS) which is responsible for translocating CagA into the host gastric epithelial cells [6]. Both CagA and the *H. pylori* T4SS have been associated with the cellular changes that mark the pathological progression of gastric cancer [7-10]. Once inside the gastric epithelial cells, CagA localises to the plasma membrane and interacts with many different host cell proteins (e.g. Abl kinases, SRC, PAR1b/MARK2 kinases, CrkII, SHP-2 protein tyrosine phosphatase), interfering with the signalling pathways that regulate cell-cell adhesion, cell growth and motility [11-18]. The CagA-mediated sustained deregulation of these pathways is believed to eventually lead to cancer. Although the cellular effects of CagA are well characterised, the structure-function relationship of this protein remains poorly understood.

**Figure 1:**
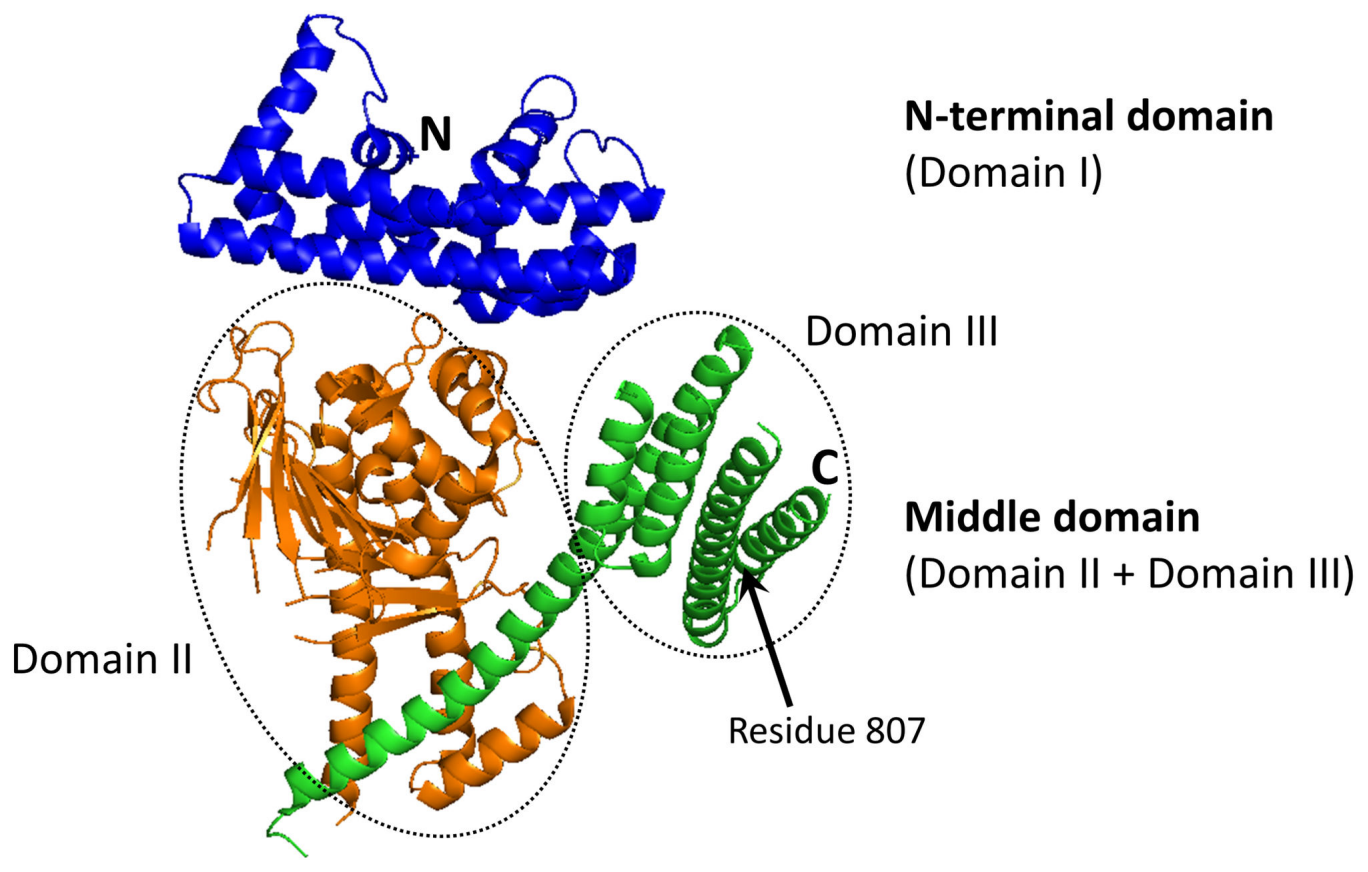
Crystal structure of the CagA region comprising the N-terminal (Domain I) and the middle (Domain II + Domain III) domains [19].

Crystallographic studies of C-terminally truncated CagA [19,20] showed that this protein has a unique fold (Figure 1). The N-terminal domain (termed Domain I by Hayashi and co-workers, who reported the first crystal structure of the truncated CagA [19]) is predominantly α-helical and shows no significant structural similarity to any other protein. The middle domain consists of two domains (termed Domain II and Domain III). Domain II comprises an extended single-layer β-sheet and two helical subdomains. This domain is rigidly connected to the second, helical Domain III by a long α-helix. No structure is available for the C-terminal region of CagA.

The first 200 amino acids of CagA were shown to be sufficient for membrane tethering [21], which suggests that the N-terminal domain is important for localisation of CagA to the phospholipid bilayer or to membrane-bound receptors. Furthermore, this domain reduces junctional and polarity defects induced by the C-terminus of CagA and is thought to play the role of an intrinsic inhibitory domain that decreases the carcinogenic potential of this protein [21]. The middle domain harbours a binding site for α_5_β_1_ integrin, the receptor that is required for CagA delivery into gastric epithelial cells [20]. In addition, it contains positively charged regions that are important for membrane binding [17,19]. The C-terminal region of CagA contains the glutamate-proline-isoleucine-tyrosine-alanine (EPIYA) sequence motifs that are the sites of phosphorylation by the Src family of protein tyrosine kinases [11,12]. Phosphorylated EPIYA segments act as binding sites for several host proteins including protein tyrosine phosphatase SHP-2 [13]. *Via* these motifs, membrane-bound phosphorylated CagA recruits SHP-2 to the plasma membrane and activates its phosphatase activity. Activated SHP-2 generates signals that lead to cell morphological changes which promote the formation of peptic ulcers and adenocarcinoma. The EPIYA repeats region was also shown to be essential for membrane tethering [22,23]. Furthermore, two putative CagA multimerisation motifs were identified in this region, which stabilise CagA-SHP-2 interaction, bind to PAR1b/MARK2 and inhibit its activity, and activate the PI3K/Akt pathway *via* Met [18,24-27]. 

In order to understand the physicochemical properties of CagA that underpin its function, we performed limited proteolysis, gel filtration, multiangle light scattering and circular dichroism (CD) analysis of the conformation, oligomeric state and thermal stability of the N-terminal, middle and EPIYA-repeats domains of CagA from *H. pylori* 26695 (termed hereafter as CagA-N, CagA-M and CagA-R, respectively). Here, we present the results of these analyses and discuss implications for the mechanism of CagA translocation and its interactions with host proteins.

## Results

### Purification of recombinant N-terminal, middle and EPIYA-repeats domains of CagA

The delineation of putative boundaries of the CagA-N (residues 22-234), CagA-M (257-880) and CagA-R (893-1001) domains was carried out as described in Materials and Methods. In order to facilitate purification by affinity chromatography, the fragments were expressed with an N-terminal His_6_ tag followed by the linker GKPIPNPLLGLDSTENLYFQ↓GIDPFT containing a TEV protease cleavage site (underlined). The results of the capture of His_6_-tagged proteins on a Ni-chelating affinity column, TEV cleavage of the His_6_ tag and subsequent purification using gel filtration are shown in File S1. The proteins were purified to >90% electrophoretic homogeneity based on Coomassie blue staining of SDS-PAGE gels, and their mass confirmed by mass spectrometry. CagA-N, CagA-M and CagA-R migrated on SDS–PAGE with an apparent molecular weight (MW) of 24, 58 and 10 kDa, respectively, which is close to the values calculated from the amino-acid sequence (25, 70 and 12 kDa). The yield of pure protein for CagA-N, CagA-M and CagA-R was approximately 0.2, 7 and 0.8 mg per l of *Escherichia coli* cell culture, respectively.

### Determination of stable core boundaries by limited proteolysis

**Figure 2:**
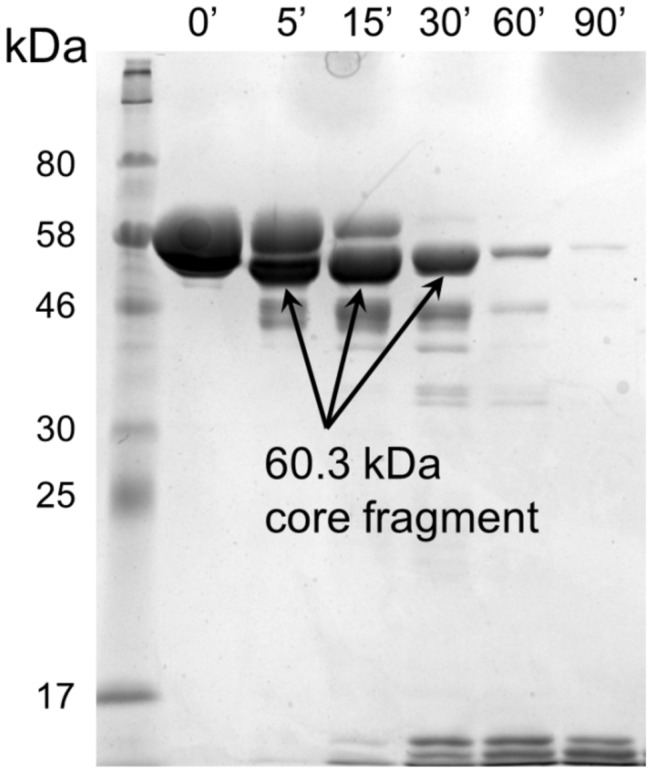
SDS-PAGE showing the time-course of digestion of CagA-M by trypsin. The arrow indicates a relatively stable core fragment CagA-M_c_.

To investigate the boundaries of each domain's stable core resistant to proteolysis, we carried out digests with trypsin and monitored the time-course of degradation by SDS-PAGE. CagA-R was fully degraded to small peptides within 30 min of digestion (Figure S2 in File S1), indicating that all the potential trypsin cleavage sites are exposed and, therefore, CagA-R is either fully unfolded or intrinsically disordered. In contrast, the time-course of degradation of CagA-M (Figure 2) demonstrated accumulation of a relatively stable fragment (the core fragment termed hereafter CagA-M_c_) before further degradation into shorter products. To determine its boundaries, the molecular mass of CagA-M_c_ was measured by matrix-assisted laser desorption ionisation-time-of-flight mass spectrometry (MALDI-TOF MS), yielding the value of 60,300 ± 100 Da. N-terminal sequencing identified the first five amino acid residues of CagA-M_c_ as GNFSK. This indicated that the N-terminus of CagA-M remained intact and defined CagA-M_c_ as fragment 267-807 with excellent agreement between the experimental and the calculated (60,288 Da) mass values. This result suggested that region 808-880 of CagA-M is flexible and accessible to protease and that the remainder has a compact and stable fold, which is consistent with the previous crystallographic studies on CagA(1-876) [19] that showed residues 825-876 were structurally disordered. This result is also in line with the previous limited trypsin proteolysis study of recombinant CagA(1-876) [19], which reported cleavage at a close albeit distinct site 829. We then cloned, expressed and purified the stable core fragment CagA-M_c_ and included it into subsequent biophysical experiments. In the tryptic digestion of CagA-N (Figure S2 in File S1), the band corresponding to intact protein (25 kDa) was present even after 3 hrs, demonstrating that this fragment is more resistant to proteolysis than CagA-M and CagA-R. Together with the fact that no accumulation of metastable fragments was observed, this suggested that fragment CagA-N folds into a single stable domain.

### Secondary structure analysis

**Figure 3:**
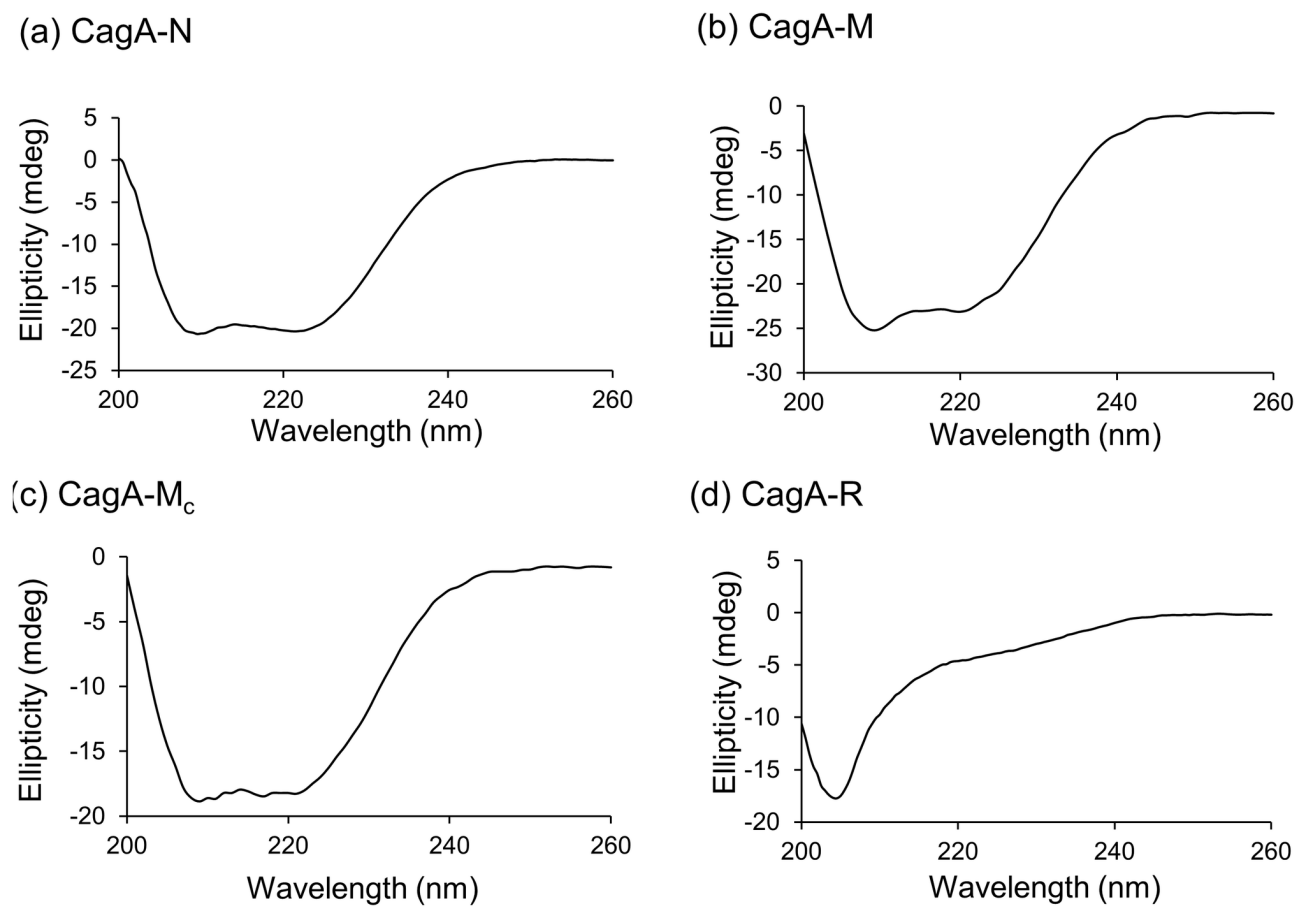
CD spectra of the recombinant CagA fragments. Ellipticity in the far-UV range (200–260 nm) is plotted for (a) CagA-N at 0.075 mg/ml, (b) CagA-M at 0.05 mg/ml, (c) CagA-M_c_ at 0.1 mg/ml and (d) Cag-R at 0.15 mg/ml.

The secondary structure of CagA-N, CagA-M, CagA-M_c_ and CagA-R was investigated using CD. The CD spectra of CagA-N, CagA-M and CagA-M_c_ (Figure 3a,b and c) exhibited double minima at approximately 208 and 222 nm, profiles which are characteristic of proteins with high α-helical content. Estimation of the α-helix and β-sheet content in the secondary structure using K2d [28] gave values that are close to those derived from the crystal structure of the truncated CagA variant [19,20] or predicted from sequence analysis using Jpred3 [29] (Table 1), indicating that the purified fragments CagA-N, CagA-M and CagA-M_c_ were folded. In contrast, the far-UV CD spectrum of CagA-R showed a single sharp minimum at around 203 nm (Figure 3d) and a relatively low ellipticity above 210 nm, indicative of poorly structured conformations with low content of secondary structure, a feature that is often attributed to intrinsically disordered proteins of premolten globule (PMG) type [30-33]. Analysis of the CD spectrum of CagA-R indicated 55% random coil and 6% helical content (the remainder was calculated to be β-sheet, although it is difficult to accurately quantify β-sheet content by CD).

**Table 1:** Secondary structure content estimated from far-UV CD spectra and predicted from sequence analysis or derived from the crystal structure [19,20].

	% α-helix, % β-sheet estimation from the CD analysis	% α-helix, % β-sheet in the crystal structure	predicted % α-helix, % β-sheet
CagA-N	58, 10	61, 0	-
CagA-M^a^	29, 15	-	41, 9
CagA-M_c_	31, 11	39, 10	-
CagA-R	6, 39	-	27, 7

^a^ Values predicted from sequence analysis rather than the crystal structure are shown as approximately 16% of the CagA-M region was not visible in the electron density maps.

### Oligomeric state of the CagA domains determined by size-exclusion chromatography coupled to multiangle light scattering analysis

To determine the oligomeric solution state, sample monodispersity and the hydrodynamic radius of the CagA domains, we carried out multiangle light scattering (MALS) and quasi-elastic light scattering (QELS, also known as dynamic light scattering, DLS) analyses coupled to size-exclusion chromatography (SEC). MALS allows shape-independent determination of the absolute molecular mass and DLS measures their hydrodynamic size. The elution of proteins from the size-exclusion column was monitored using inline UV/Vis, MALS and DLS detectors. 

**Figure 4:**
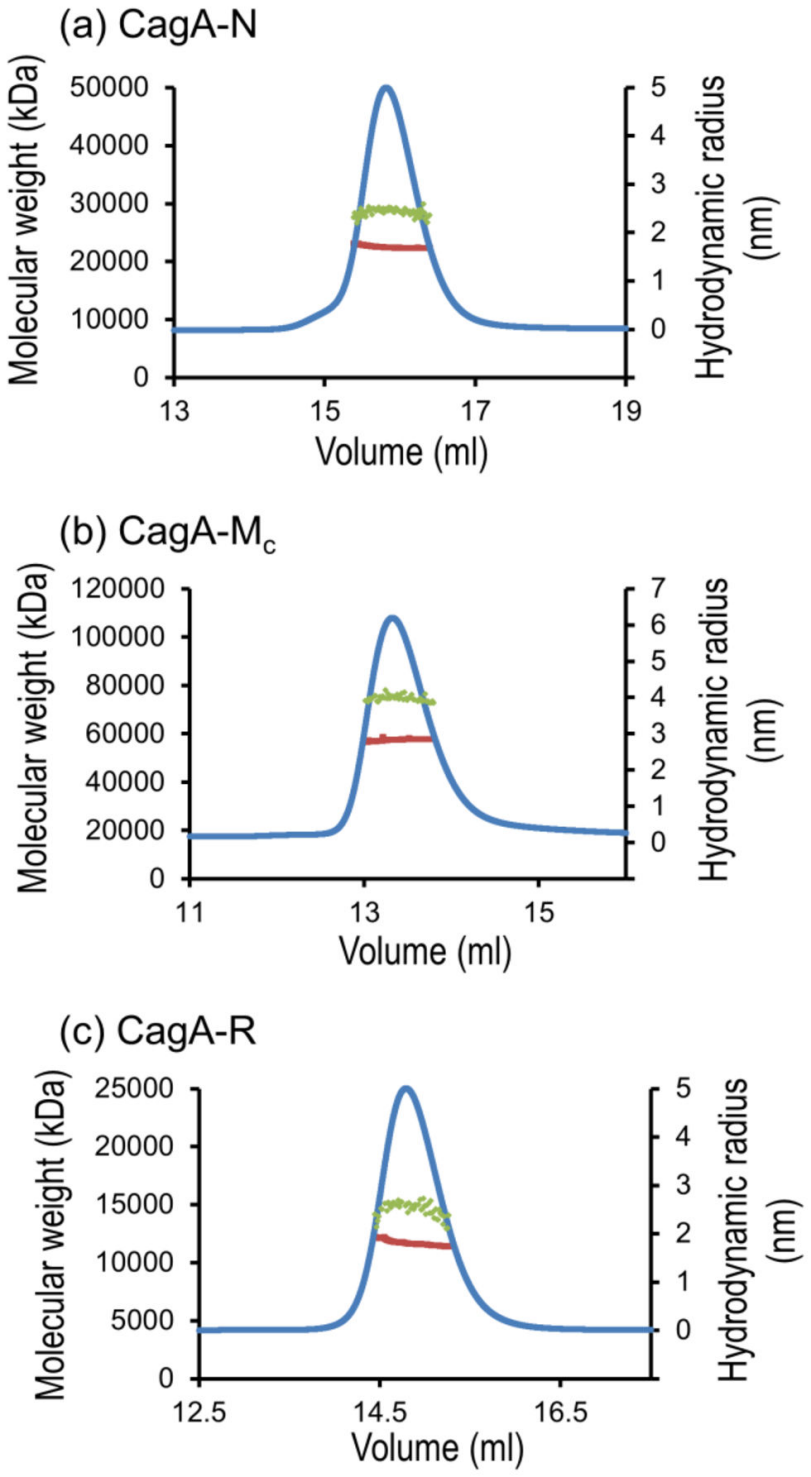
SEC and molecular weight (MW) and hydrodynamic radius determination of CagA-N (a), CagA-M_c_ (b) and CagA-R (c). Green dots superimposed on the peak indicate the MW as shown on the left-hand y-axis. Red dots represent the hydrodynamic radius calculated over the central portion of the elution peak (shown by UV trace in blue). The hydrodynamic radius values are shown on the right-hand y-axis.

CagA-N eluted as a single, monodisperse peak with the derived MW value of 22.5±2.3 kDa (Figure 4a; we estimated the accuracy of the weight determination as approximately 10% from the quality of the BSA standard). This value is consistent with a monomer. The apparent hydrodynamic radius of the particles in this peak was 2.5±0.3 nm (Figure 4a), which is in excellent agreement with the value of the hydrodynamic radius calculated using the 3D coordinates of the N-terminal CagA domain (region 24-221) in the crystal [19] (2.5 nm).

Both CagA-M and CagA-M_c_ also eluted as a single, monodisperse peak (Figure 4b; data is only shown for CagA-M_c_). The derived MW of CagA-M and CagA-M_c_ was 76.0±7.6 kDa and 57.5±5.8, respectively, indicating that both fragments are monomeric in solution. Analysis of light scattering data allowed us to calculate the values of the apparent hydrodynamic radius for CagA-M (4.5±0.5 nm) and CagA-M_c_ (4.0±0.4 nm). Although the structure of the full fragment Cag-M or CagA-M_c_ is not known due to disorder of the region 222-302 in the crystal, we noted that the hydrodynamic radius of the fragment 267-807 (CagA-M_c_, 4.0 nm) in solution is close to that calculated for the slightly shorter region 303-807, the structure of which is available (3.7 nm).

The elution profile of CagA-R, the three-dimensional structure of which is not yet known, showed a single peak with a median MW of 11.6±1.2 kDa and a hydrodynamic radius of 2.6±0.3 nm. The MW is in close agreement with the calculated weight of a monomer (12 kDa). The expected hydrodynamic radius for a 12 kDa globular protein is around 1.8 nm (for example, the hydrodynamic radius of 12.4 kDa spherical protein cytochrome C is 1.78 nm [34,35]). The hydrodynamic radius calculated for CagA-R in solution (2.6 nm) is significantly larger and corresponds to a globular protein of approximately 28 kDa which is two times its calculated weight. We verified this result by calibrating the column with spherical molecular weight standards. Cytochrome C, a protein with a mass close to that of CagA-R, eluted at 16 ml. CagA-R eluted significantly earlier in a volume of 14.9 ml which is close to that of 25-kDa globular domain CagA-N (Figure 4c). Given the monomeric state of CagA-R, a significantly smaller elution volume and a larger hydrodynamic radius for a protein of a given mass is indicative of the extended structural state (i.e., disorder). From the value of the apparent MW, the type of disorder, such as molten globule-, PMG-, or random-coil-type, can be determined [33]. Compact PMG type intrinsically disordered proteins elute at an apparent molecular weight twice its real value, whereas more extended, random-coil type disordered proteins elute at an apparent MW that is four to six times its real value [33,36]. Our gel filtration studies therefore suggested that CagA-R is a PMG type intrinsically disordered protein.

### Stability of isolated domains against thermal denaturation

**Figure 5:**
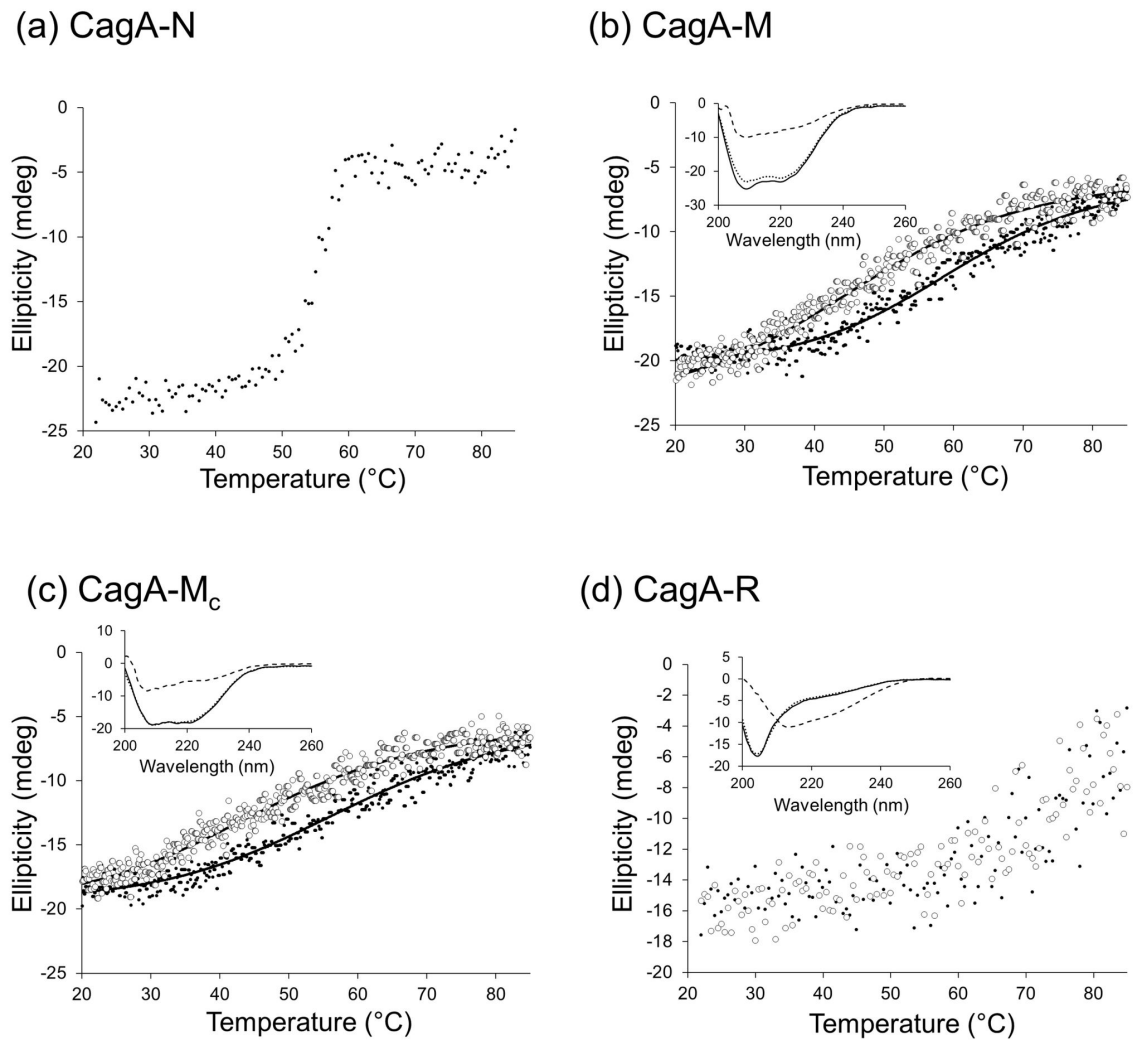
Thermal unfolding and refolding transitions of CagA domains monitored by far-UV CD. The unfolding data is shown with black dots for CagA-N (a), CagA-M (b), CagA-M_c_ (c) and CagA-R (d). Unfolding was reversible for CagA-M, CagA-M_c_ and CagA-R; the refolding data is shown with open circles. The insets show the corresponding CD spectra for CagA-M (b), CagA-M_c_ (c) and CagA-R (d) for the native (solid line), unfolded (85 °C, dashed line) and refolded (dotted line) states. The low signal-to-noise ratio for the CagA-*R* *spectrum* reflects the fact that the scan for this fragment was performed at lower wavelengths (205 nm rather than 222 nm), where the absorbance is inherently higher and thus the data is collected at a higher dynode voltage.

We examined the thermodynamic stability of CagA-N, CagA-M, CagA-M_c_ and CagA-R by CD. The results of the variable temperature far-UV CD spectroscopy experiments indicated that CagA-N undergoes a thermal transition between 50 and 60°C and is essentially denatured at higher temperatures (Figure 5a). The apparent midpoint temperature of the unfolding transition of CagA-N (T_*m*_^app^), that is defined as the inflection point of the sigmoidal melting curve at 222 nm and represents the temperature at which 50% of the protein is unfolded, was 54°C. The unfolding of CagA-N was irreversible due to aggregation of thermally denatured protein, thus precluding the direct measurement of its stability. 

CagA-M and CagA-M_c_ exhibited reversible sigmoidal melting curve profiles at 222 nm (Figure 5b and c) that could be approximated using a simple two-state unfolding model. Heating CagA-M and CagA-M_c_ to 85°C resulted in a loss of secondary structure, consistent with denaturation (Figure 5b and c). Upon cooling, the secondary structures of the heat-treated proteins returned to a state similar to, but not identical with that of the unheated controls. Thus, the CD spectra also confirmed that the majority of the heat-induced structural changes in CagA-M and CagA-M_c_ were reversible. The reversibility of their thermal transitions allowed a thermodynamic analysis of unfolding equilibria. We determined the van't Hoff enthalpy (ΔH) and entropy (ΔS) of unfolding, the midpoint of the unfolding transition (T_*m*_) and the free energy of unfolding (ΔG) (Table 2). Compared to CagA-N, the unfolding of CagA-M and CagA-M_c_ was significantly more gradual, over a very broad temperature range (>35°C), which is indicative of low cooperativity of the melting process. This observation is consistent with the previous structural studies on CagA [19,20] which showed that although residues 261-829 form a protease-resistant structural unit, their fold is a dimodular structure consisting of domains Domain II (303-644) and Domain III (720-824) rigidly connected by a long α-helix 648-708.

**Table 2:** Thermodynamic parameters obtained from CD for the unfolding of CagA-M and CagA-M_c_ (1 kcal = 4.18 kJ).

	van't Hoff enthalpy of unfolding (ΔH), kcal/mol	Entropy (ΔS) of unfolding (37°C), cal/ K·mol	The midpoint of the unfolding transition (T_*m*_), °C	Free energy of unfolding (ΔG, 37°C), kcal/mol
CagA-M	20.4	61.4	59.7	1.4
CagA-M_c_	16.4	49.7	57.6	1.0

Heating CagA-R from 22 to 85°C resulted in a shift of the CD spectrum by increasing the signal at around 200 nm and decreasing at 222 nm (Figure 5d). The decrease in signal at 222 nm upon heating is characteristic of left-handed, extended helical conformation termed the poly-l-proline type II (PPII) helix [37-39], suggesting that CagA-R contains regions in PPII conformation. Since the CD spectra of native and unfolded CagA-R showed a minimum at around 203 and 212 nm, respectively, CagA-R unfolding was followed as a function of temperature increase by monitoring the signal amplitude at 205 nm. The forward and reverse melting curves for CagA-R were not sigmoidal, as for typical folded proteins, but progressive, indicating no obvious structural cooperativity of unfolding (Figure 5d). The CD spectra of CagA-R prior to heating and after cooling back to 22°C were very similar (Figure 5d), indicating that the thermal unfolding is reversible. 

## Discussion

CagA is a multifunctional effector protein that is translocated by *H. pylori* by means of its T4SS. The proposed elementary molecular events that constitute the mechanism of CagA action include recognition by the cytoplasmic chaperone CagF, translocation through the T4SS in an unfolded form, refolding following translocation, binding to the phospholipid bilayer of the membrane and interactions with around 20 different host proteins. Although the crystal structure of part of this protein is available, little is known about the thermodynamic properties of its individual domains that underpin its function. Here, we have investigated the domain organisation of CagA, and the conformation, oligomeric state and stability of the N-terminal, middle and EPIYA-repeats domains by using genetic engineering, limited proteolysis, gel filtration, SEC/MALS and CD techniques.

### Isolated recombinant N-terminal, middle and EPIYA-repeats domains of CagA are folded and monomeric in solution

It has been shown that CagA dimerises *in vivo* when bound to cellular proteins Par1b/MARK2 [25,26] and SHP2 [24,25] and that the dimerisation is important for CagA function. Recently, regions 946-961 and 981-995 were identified as putative CagA multimerisation motifs (CM motifs) [18,24-26]. However, it remains unknown whether CagA can dimerise on its own or if its observed apparent oligomerisation *in vivo* is a consequence of binding to multimeric partner proteins. The results of our SEC/MALS experiments on highly pure, properly folded N-terminal (residues 22-234), middle (257-880) and EPIYA-repeats (893-1001) CagA domains showed that they behave as monodisperse monomers in solution under our experimental conditions. This result is consistent with the previous studies of recombinant CagA fragments under different experimental conditions [19] and favours the hypothesis that CagA oligomerisation may be potentiated by interaction with multimeric partner proteins. SHP-2, for example, has been hypothesised to engage two CagA molecules simultaneously *via* its N-terminal and C-terminal SH2 domains [13]. 

### The N-terminal fragment 22-234 (CagA-N) forms a single compact domain that does not refold spontaneously following heat denaturation

A number of observations from this study indicated that the fold of the isolated N-terminal fragment 22-234 (CagA-N) is very close to that found in the crystal structure. Firstly, CD analysis of CagA-N in solution revealed that its predominantly α-helical secondary structure content is close to that observed in the crystal. Secondly, temperature denaturation studies showed that the transition from native to denatured state was very steep, occurring over a narrow range of 10°C, indicative of a highly cooperative unfolding reaction. Together with the fact that CagA-N was more resistant to proteolysis than CagA-M and CagA-R, this indicated that CagA-N forms a single, folded, compact domain with multiple tertiary contacts between α-helices, as seen in the crystal structure of the N-terminal CagA domain. Furthermore, the value of the hydrodynamic radius of CagA-N calculated from the QELS analysis (2.5 nm) matched that calculated for the crystal structure.

The apparent melting temperature of CagA-N was around 54°C which suggested that this domain is stable under physiological (37°C) conditions. Interestingly, our novel thermal melting data showed that unlike CagA-M and CagA-R, the unfolding of CagA-N was irreversible due to aggregation of thermally denatured protein. This has implications for the mechanism of translocation of CagA *via* T4SS. The secretion signal of most of the characterised T4SS substrates (including CagA) in different bacterial species is located at the C-terminus [40,41]. Although much remains unknown regarding the detailed mechanism of the protein secretion through T4SS, it is believed that the substrates are threaded through the secretion channel in an unfolded state and that the polypeptide chain emerging from the channel refolds spontaneously, without any chaperone assistance. Our observation that the N-terminal domain of CagA does not refold by itself therefore suggested that its folding outside of the bacterial cell likely requires interactions with the remainder of the protein.

Since the secretion signal that targets the protein to the T4SS translocation channel is located at the C-terminus, it is likely that the secreted protein, such as CagA, is threaded C-terminus first and the N-terminus emerges from the channel last. Our studies suggested that the C-terminal part of CagA, including the EPIYA region and the middle domain, would spontaneously fold as it emerges, and likely serve as the intramolecular chaperone that facilitates folding of the N-terminal domain.

### The EPIYA-repeats region 983-1001 exists in an intrinsically disordered premolten globule state

The EPIYA-repeats region of CagA is of great functional importance as it contains the EPIYA motifs that serve as the sites of phosphorylation and the CM motifs that potentiate CagA interaction with PAR1b/MARK2 and SHP-2. In addition to the latter two, CagA interacts with around 20 different host cell proteins [14], with most interactions being phosphorylation-dependent and thus involving the EPIYA-repeats region. It has been proposed that such versatility and promiscuity of function is underpinned by the natural structural flexibility of the C-terminal CagA region containing the EPIYA-repeats [19]. Although the intrinsically disordered nature of the C-terminus of CagA has been established in previous studies [18,19], the type of disorder (coil-like or PMG [42]) was not known. Our analysis of the conformational properties of the EPIYA-repeats region 983-1001 based on measurements of hydrodynamic radii allowed us to designate it as PMG. It satisfied the following physicochemical criteria for this state [33]: (1) rapid and complete proteolysis under conditions where typical folded proteins show accumulation of metastable fragments; (2) very low secondary structure content detected by far-UV CD; (3) larger hydrodynamic radius determined by SEC/MALS (elutes at an apparent molecular weight approximately twice its real value); (4) heat stability and (5) absence of a cooperative transition on a thermal melting curve. These results are in line with previous NMR characterisation of the CagA fragment 877-1026, which reported the spectrum typical for an intrinsically disordered polypeptide [19]. They are also in agreement with the crystallographic study of the complex between Par1b/MARK2 and CagA fragment 893-1004 [18] where most of the CagA 893-1004 polypeptide chain was not seen in the electron density maps due to conformational disorder.

Intrinsic disorder has been predicted to be a common feature of the bacterial EPIYA motifs [43]. Do all EPIYA-containing domains exist in a PMG rather than coil-like state? Previous measurements of the hydrodynamic radius of the natively unfolded 20-kDa C-terminal region of the enteropathogenic *E. coli* translocated intimin receptor (Tir) protein, which contains two EPIYA-related motifs, yielded the value of 3.9 nm for a monomer [44]. This radius corresponds to a globular protein of approximately 47 kDa [35], which is approximately two times the calculated weight. Thus, similar to CagA, the EPIYA-containing domain of a different bacterial effector protein, Tir, exists in a PMG rather than random-coil state. It is anticipated that as more bacterial EPIYA effectors are identified and characterised [43], further commonalities in type of disorder will be revealed.

Unlike intrinsic coils, premolten globules exhibit some amount of residual secondary structure [33]. Our CD analysis showed for the first time that the disordered CagA-R domain contains regions in PPII helix conformation. Although the boundaries of such regions within CagA-R cannot be estimated using existing secondary structure prediction algorithms, this observation has important implications for the function of the C-terminus of CagA. PPII helices are abundant in intrinsically disordered regions of proteins and are one of the most widespread binding motifs for partner proteins or nucleic acids [39]. One distinctive structural feature of PPII helices that appears to be important in protein-protein interactions is the presence of exposed and non-satisfied main chain hydrogen bonding donor and acceptor groups that are free to establish intramolecular interactions [45]. The second feature is the exposed hydrophobic side chains that can be recognised by hydrophobic patches present on the partner protein. The observation that CagA-R contains regions in the PPII conformation is therefore consistent with the previously identified role of this domain as the main site of interactions with host proteins [43]. Furthermore, due to their distinct structural properties, the PPII helical regions may be a factor that contributes to incredible versatility and promiscuity of the interactions mediated by this domain. PPII helices are highly hydrated and, upon binding to a partner protein, tend to preserve at least part of their hydration shell, so that the binding interface contains many water-mediated, rather than direct, hydrogen bonds [46]. Water at the interface of a complex can serve as an adaptor to facilitate promiscuous recognition of diverse proteins. 

The versatility of interactions mediated by the disordered C-terminus of CagA gives rise to the notion that it may mimic eukaryotic scaffold proteins and act as a pathogenic scaffold that recruits multiple host proteins potentiating oncogenic signalling [43,47]. Similar to CagA, the eukaryotic scaffold proteins, such as Grb2-associated binder (Gab), insulin receptor substrate (IRS), downstream of kinase (DOK) proteins, and Crk-associated substrate (Cas), consist of one or two folded N-terminal domains, followed by a predominantly disordered C-terminal tail region which serves as a binding site for multiple proteins [48]. It has been shown that, like CagA, the C-terminal tails of Gab and Cas contain regions in PPII conformation that are essential for their binding to Src and Grb2, respectively [49,50]. This raises the interesting question of whether the presence of PPII helical regions in the disordered EPIYA-containing domains of bacterial effector proteins is a conserved feature.

## Materials and Methods

### Delineation of putative structural domain boundaries of CagA

The domain boundaries of CagA from *H. pylori* strain 26695 (Genbank ID: AAD07614) were first predicted using DomPro [51] as 1 - 236 (N-terminal domain), 237 - 891 (middle domain) and 892 - 1186 (C-terminal domain). These boundaries were close to those delimited by the previous limited proteolysis studies by Hayashi and co-workers (1-260, 261-876 and 877-1186 [19]). We then predicted the secondary structure using the Jpred3 server (http://www.compbio.dundee.ac.uk/www-jpred/) [29] and eliminated unstructured N- and C-termini. This gave a set of three fragments, for which we tested recombinant expression in *E. coli*: Cag-N, comprising amino acid residues 22-234, CagA-M (257-880) and CagA-C (893-1171). The expression levels of CagA-C were too low for structural studies, likely due to the presence of the C-terminal secretion signal sequence that may prevent spontaneous folding of the C-terminus in the cytoplasm [40,41]. However, Nesic et al. have previously purified a stable, smaller fragment 893-1004 of the C-terminal region for co-crystallisation with Par1b/MARK2 [18]. This region contains all three EPIYA repeats. We refined the boundaries of this repeats domain (termed CagA-R) to 893-1001 so that no predicted secondary structure elements were interrupted.

### Cloning, recombinant expression and purification of CagA fragments

The coding sequences for CagA-N (residues 22-234), CagA-M (257-880), CagA-M_c_ (267-807) and CagA-R (893-1001) were PCR-amplified from genomic DNA of strain 26695 of *H. pylori* using *Pfu* DNA polymerase (Stratagene). The amplified genes were cloned into the pET151/D-TOPO vector using the TOPO cloning kit (Invitrogen) to produce expression vectors that contained an N-terminal His_6_ tag followed by a TEV protease cleavage site. The expression clones were confirmed by DNA sequencing and transformed into *E. coli* strain BL21 DE3 (Novagen). Cells were grown in LB medium containing 50 mg/l ampicillin at 37 °C until an OD_600_ of 0.8 was reached, at which point protein overexpression was induced by adding 1 mM IPTG and growth continued for a further 3 hrs. The cells were then harvested by centrifugation at 6,000 *g* for 15 min at 4 °C.

For the purification of each fragment, the cells were resuspended in buffer A (20 mM sodium phosphate pH 7.4, 200 mM NaCl and 1 mM PMSF) and lysed using the EmulsiFlex-C5 high-pressure homogeniser (Avestin). Cell debris was removed by centrifugation at 12,000 *g* for 30 min at 4 °C. The supernatant was collected and clarified by ultracentrifugation at 105,000 *g* for 20 min at 4 °C. NaCl and imidazole were then added to the supernatant to the final concentrations of 500 mM and 10 mM, respectively, after which the supernatant was loaded onto a 5-ml Hi-Trap Chelating HP column (GE Healthcare) pre-washed with buffer A containing 500 mM NaCl. The column was washed with 20 column volumes of buffer B (20 mM sodium phosphate pH 7.4, 500 mM NaCl, 40 mM imidazole) and protein was eluted with buffer B containing 500 mM imidazole. The N-terminal His_6_ tag was cleaved off with His_6_-TEV protease (Invitrogen) overnight at 4 °C whilst dialysing the sample against buffer C (50 mM Tris/HCl pH 8.0, 0.5 mM EDTA, 2 mM DTT, 200 mM NaCl, 1%(v/v) glycerol). NaCl and imidazole were then added to the sample to the final concentrations of 500 mM and 15 mM, respectively, and the TEV protease and uncleaved protein were removed over a Hi-Trap Chelating HP column. The flow-through was concentrated to 4 ml in a VivaSpin 10,000 Da cut-off concentrator and loaded onto a Superdex 75 HiLoad 26/60 gel-filtration column (GE Healthcare) equilibrated with buffer D (30 mM NaAc pH 4.6, 200 mM NaCl). The peak fractions were pooled, dialysed overnight against 50 mM Tris/HCl pH 8.0, mixed with glycerol (20%(v/v)) and snap-frozen in liquid nitrogen for storage. Protein concentration was determined using the Bradford assay [52]. An analytical size-exclusion experiment was performed on a Superdex 200 10/300 HiLoad gel filtration column (GE Healthcare) pre-equilibrated with buffer D flowing at 0.4 ml/min and calibrated with globular (quasispherical) proteins using a Gel Filtration Markers Kit (MWGF70, Sigma-Aldrich).

### Limited proteolysis and identification of the protein fragments

Limited proteolysis by trypsin was performed in a buffer containing 50 mM Tris/HCl pH 8.5 and 5 mM CaCl_2_ at 30 °C and a protein concentration of 1 mg/ml. In a typical reaction sequencing-grade trypsin (Roche) was added to the protein solution at a ratio of protease to protein of 1:300 (w/w). At various times aliquots were removed for subsequent analysis where the reaction was stopped either by adding SDS-PAGE loading dye and incubating the sample at 30 °C for 30 min (for electrophoretic analysis) or by adding 5%(v/v) trichloroacetic acid (for mass spectrometry).

Proteins and their tryptic fragments were analysed by means of MALDI-TOF MS. A 1 μl aliquot of each sample was mixed with 1 μl of a matrix solution containing 50%(v/v) acetonitrile, 0.3%(v/v) trifluoroacetic acid and 10 mg/ml sinapinic acid, spotted on the MALDI sample plate and allowed to dry. Molecular mass analysis was performed using a mass spectrometer Voyager DE/PRO (PerSeptive Biosystems) at Osaka University and a Proteomics Analyser 4700 (Applied Biosystems) at Monash University.

For the N-terminal sequencing of the stable core fragment, proteins in SDS/polyacrylamide gel were transferred onto a polyvinylidene difluoride (PVDF) membrane (Bio-Rad) with a Hoefer transblotting apparatus and stained for 5 min with 0.025%(w/v) Coomassie brilliant blue in 40%(v/v) methanol. After destaining the PVDF membranes for 10 min with 50%(v/v) methanol, the band of interest was cut out and subjected to N-terminal amino acid sequence analysis at the Institute for Protein Research (Osaka University) or at the Monash University Biomedical Proteomics Facility.

### Far-UV CD spectroscopy

Far-UV CD spectra were recorded at protein concentrations of 0.075 (CagA-N), 0.05 (CagA-M), 0.1 (CagA-M_c_) and 0.15 (CagA-R) mg/ml at 20 °C on a JASCO J-810 spectrapolarimeter over the wavelength range 200–260 nm with the scan rate of 20 nm/min, data pitch of 0.5 nm and path-length of 0.2 nm. Spectra were recorded five times and averaged. All measurements were corrected for solvent (buffer D) contributions and smoothed using the Savitsky-Golay algorithm with a radius of 25. The percentage of secondary structure was calculated by deconvoluting the CD spectra using the program K2d from the DichroWeb CD secondary structure server [28] (http://dichroweb.cryst.bbk.ac.uk/html/home.shtml).

Thermal denaturation measurements were performed at a protein concentration of 0.075-0.15 mg/ml over a temperature range of 20 to 85 °C using a 0.2-cm path-length thermostated quartz cell. Data was collected at a heating/cooling rate of 2 °C/min. The CD signal was continuously monitored every 0.5 °C at 222 nm for CagA-N and CagA-M and 205 nm for CagA-R. After heating to 85 °C, the protein samples were cooled to 20 °C. The far-UV CD spectra were recorded over the wavelength range 190–260 nm at the start point (20 °C), highest temperature point (85 °C), and the cooling end point (20 °C). All the spectra were corrected for solvent contribution at increasing temperatures. The data was fitted to the Gibbs-Helmholtz equation that describes the folding as a function of temperature using the two-state unfolding model as described in [53]. The fitting model assumed a temperature-independent enthalpy. Thermal denaturation of CagA-N was irreversible due to the protein aggregation at high temperatures, and therefore the apparent thermal denaturation temperature (T_*m*_^app^) is reported.

### SEC and MALS/QELS analysis

The proteins were concentrated to 5-7 mg/ml. A 50 µl sample was loaded onto a Superdex 200 10/300 HiLoad gel filtration column pre-equilibrated with buffer D flowing at 0.4 ml/min. The eluate was passed through an in-line DAWN HELEOS II laser photometer, an Optilab T-rEX differential refractive index detector (Wyatt Technologies) and a dynamic light scattering detector (WyattQELS, Wyatt Technologies). A bovine serum albumin (BSA) standard was used to normalise the MALS detectors. Data was analysed in ASTRA 6.0 (Wyatt), with a value for the refractive index increment (*dn*/*dc*)_protein_ of 0.185 ml/g. The column was calibrated using the following molecular weight standards in a Gel Filtration Markers Kit (MWGF70, Sigma-Aldrich): blue dextran (2000 kDa); albumin (66 kDa); carbonic anhydrase (29 kDa); cytochrome C (12.4 kDa); and aprotinin (6.5 kDa). Theoretical calculations of the hydrodynamic radius from the crystal structure were carried out as described by Ortega et al. [54] using HYDROPRO v. 10 (http://leonardo.inf.um.es/macromol/programs/hydropro/hydropro.htm).

## Supporting Information

File S1 **File includes Figures S1 and S2.** **Figure S1.** SDS-PAGE analysis of expression and purification of CagA domains. Lanes 1–3 show induction and solubility in BL21 (DE3) cells: (1) total protein from uninduced cells; (2) total protein from induced cells; (3) soluble protein fraction from induced cells. Lanes 4-8 and w show steps in purification: (4) unbound protein fraction after flowing cell lysate through a Ni-chelating column; (w) flow-through in the wash step with buffer B; (5) eluate from the Ni-chelating column after wash; (6) the product of TEV cleavage (7) the product of TEV cleavage passed through the Ni-chelating column; (8) pooled fractions after gel filtration chromatography. **Figure S2.** SDS-PAGE showing the time-course of digestion of CagA-N (a) and CagA-R (b) by trypsin.
(PDF)
