# Supplementary material for: Conformational Analysis of Isolated Domains of Helicobacter pylori CagA
Source: PLoS One. 2013 Nov 1;8(11):e79367. doi: 10.1371/journal.pone.0079367 (PMC3815135; doi:10.1371/journal.pone.0079367)
Supplement: File S1 — File includes Figures S1 and S2. Figure S1. SDS-PAGE analysis of expression and purification of CagA domains. Lanes 1–3 show induction and solubility in BL21 (DE3) cells: (1) total protein from uninduced cells; (2) total protein from induced cells; (3) soluble protein fraction from induced cells. Lanes 4-8 and w show steps in purification: (4) unbound protein fraction after flowing cell lysate through a Ni-chelating column; (w) flow-through in the wash step with buffer B; (5) eluate from the Ni-chelating column after wash; (6) the product of TEV cleavage (7) the product of TEV cleavage passed through the Ni-chelating column; (8) pooled fractions after gel filtration chromatography. Figure S2. SDS-PAGE showing the time-course of digestion of CagA-N (a) and CagA-R (b) by trypsin. (PDF) [file pone.0079367.s001.pdf]

Supplementary data

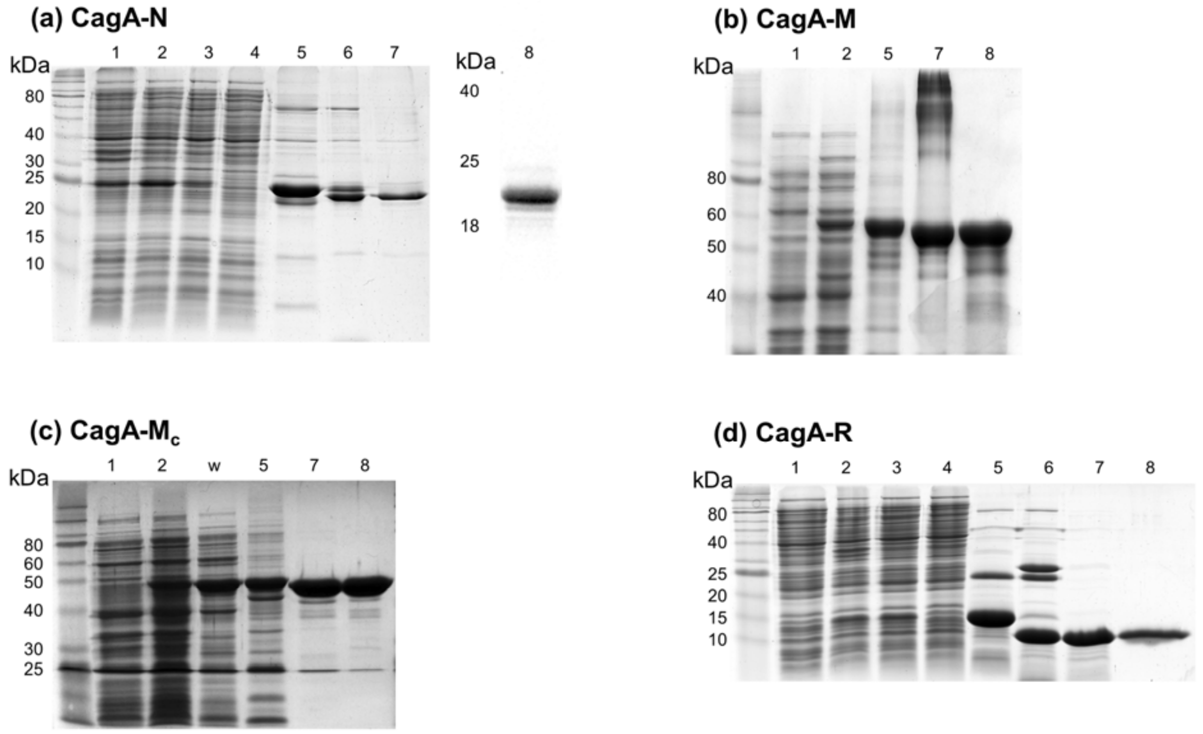

Fig. S1. SDS-PAGE analysis of expression and purification of CagA domains. Lanes 1–3 show induction and solubility in BL21 (DE3) cells: (1) total protein from uninduced cells; (2) total protein from induced cells; (3) soluble protein fraction from induced cells. Lanes 4-8 and w show steps in purification: (4) unbound protein fraction after flowing cell lysate through a Ni-chelating column; (w) flow-through in the wash step with buffer B; (5) eluate from the Ni-chelating column after wash; (6) the product of TEV cleavage (7) the product of TEV cleavage passed through the Ni-chelating column; (8) pooled fractions after gel filtration chromatography.

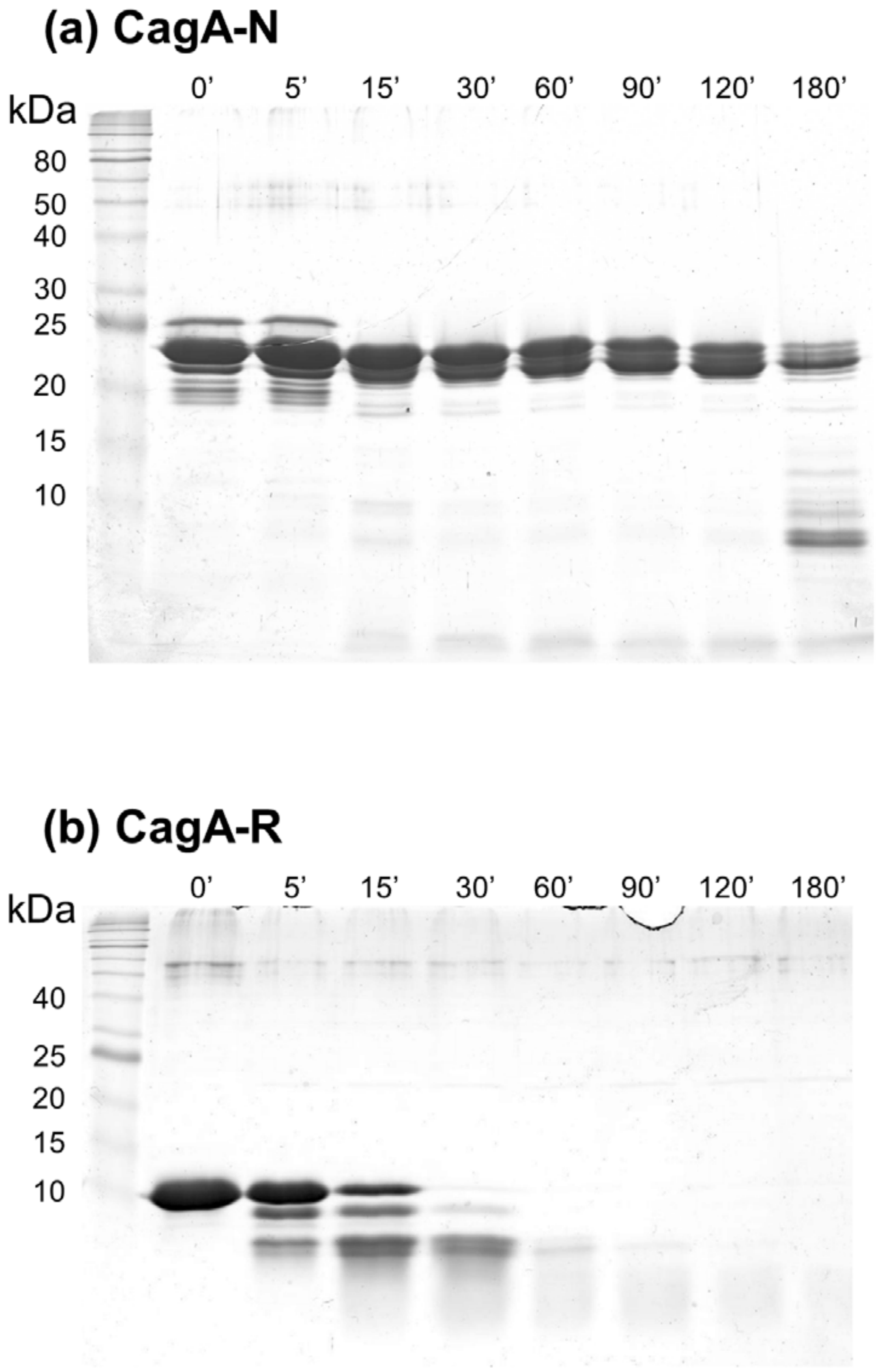

Fig. S2. SDS-PAGE showing the time-course of digestion of CagA-N (a) and CagA-R (b) by trypsin.
